# Prevalence and determinants of menstrual regulation among ever-married women in Bangladesh: evidence from a national survey

**DOI:** 10.1186/s12978-019-0785-7

**Published:** 2019-08-14

**Authors:** Juwel Rana, Kanchan Kumar Sen, Toufica Sultana, Mohammad Bellal Hossain, Rakibul M. Islam

**Affiliations:** 1 South Asia Institute for Social Transformation (SAIST), Dhaka, Bangladesh and Environmental and Occupational Health Sciences, EHESP French School of Public Health, Paris, France; 2 Department of Statistics, University of Dhaka, Dhaka, Bangladesh; 3 Department of Sociology, University of Saskatchewan, Saskatoon, Canada; 4 Department of Population Sciences, University of Dhaka, Dhaka, Bangladesh; 5 Department of Epidemiology and Preventive Medicine, Monash University, Melbourne, Australia

**Keywords:** Menstrual regulation, Prevalence, Determinants, Maternal health, Bangladesh, regulación menstrual, prevalencia, determinantes, salud materna, Bangladesh

## Abstract

**Background:**

Despite the remarkable reduction of maternal mortality, unsafe and untimely menstrual regulation (MR) remains a major maternal health problem in Bangladesh. This study aimed to determine the prevalence and identify determinants of MR among ever-married women in Bangladesh.

**Methods:**

Data for this study have been extracted from Bangladesh Demographic and Health Survey (BDHS) 2014. The survey followed a two-stage stratified sampling procedure and the study used a sub-sample of 8084 ever-married women aged 15 to 49 years extracted from survey sample of 17,863. Univariate and multivariate mixed-effect logistic regression analyses were used to identify risk factors for MR accounting for potential between-clusters variations.

**Results:**

The weighted prevalence of MR was 12.3% (95% CI: 11.1–13.4%) among (991/8084) ever-married women. Women were less likely to have MR if they were from Chittagong (AOR 0.74, 95% CI: 0.57–0.96; *p* = 0.026) and Sylhet (AOR 0.53, 95% CI: 0.36–0.77; *p* = 0.001) divisions. Women were more likely to have MR if they were from high (AOR 1.47, 95% CI: 1.18–1.83; *p* = 0.001) and the highest (AOR 1.62, 95% CI: 1.27–2.05; *p* < 0.001) socioeconomic status (SES) group; being employed (AOR 1.35, 95% CI: 1.16–1.56; *p* < 0.001), having one or two children (AOR 1.73, 95% CI: 1.24–2.40: *p* = 0.001) and ≥ 3 children (AOR 2.56, 95% CI: 1.82–3.58; *p* < 0.001), and having membership of non-government organization (NGO) (AOR 1.18, 95% CI: 1.02–1.38; *p* = 0.030).

**Conclusion:**

MR is prevalent among Bangladeshi women and independently associated with geographic location, SES, parity, employment and NGO membership status. Health policy should prioritize in reducing spatial and socioeconomic inequalities in relation to MR services by ensuring accessibility and availability of MR services, especially in suburban divisions. Furthermore, abortion should be legalized in Bangladesh that will ultimately reduce the morbidity and mortality associated with unsafe abortion.

## Spanish abstract

**Antecedentes:**

a pesar de la notable reducción de la mortalidad materna, la regulación menstrual (RM) insegura y prematura sigue siendo un problema importante de salud materna en Bangladesh. El objetivo de este estudio fue determinar la prevalencia e identificar los determinantes de la RM en mujeres que han estado casadas en Bangladesh.

**Métodos:**

los datos para este estudio se extrajeron de la Encuesta Demográfica y de Salud de Bangladesh (BDHS) de 2014. La encuesta siguió un procedimiento de muestreo estratificado en dos etapas y el estudio utilizó una sub-muestra de 8.084 mujeres casadas de 15 a 49 años de edad extraídas de la muestra de la encuesta de 17.863. Se utilizaron análisis de regresión logística de efectos mixtos univariados y multivariados para identificar los factores de riesgo de la RM tomando en cuenta las variaciones potenciales entre los grupos.

**Resultados:**

La prevalencia ponderada de la RM fue de 12.3% (IC 95%: 11.1–13.4%) entre (991/8.084) mujeres casadas. Las mujeres tenían menos probabilidades de tener una RM si eran de Chittagong (AOR 0.74, IC 95%: 0.57–0.96; *p* = 0.026) y Sylhet (AOR 0.53, IC 95%: 0.36–0.77; *p* = 0.001). Las mujeres eran más propensas a tener una RM si pertenecían al estatus socioeconómico (ESE) alto (AOR 1.47, IC 95%: 1.18–1.83; *p* = 0.001) y más alto (AOR 1.62, IC 95%: 1.27–2.05; *p* < 0.001); estar empleada (AOR 1.35, IC 95%: 1.16–1.56; *p* < 0.001), tener uno o dos hijos (AOR 1.73, IC 95%: 1.24–2.40: *p* = 0.001) y ≥ 3 niños (AOR 2.56, 95% IC: 1.82–3.58; *p* < 0.001), y pertenecer a una organización no gubernamental (ONG) (AOR 1.18, IC 95%: 1.02–1.38; *p* = 0.030).

**Conclusión:**

la RM prevalece entre las mujeres de Bangladesh y está asociada de forma independiente con la ubicación geográfica, ESE, paridad, empleo y condición de miembro de una ONG. Las políticas de salud deben priorizar la reducción de las desigualdades espaciales y socioeconómicas en relación con los servicios de RM, garantizando la accesibilidad y disponibilidad de los servicios de RM, especialmente en las divisiones suburbanas. Además, el aborto debería legalizarse en Bangladesh, lo que en última instancia reducirá la morbilidad y la mortalidad asociadas con el aborto inseguro.

## Plain English summary

Despite the remarkable reduction of maternal mortality, the incidence of abortion and proportion of abortion-related maternal deaths has increased from 2010 to 2016 in Bangladesh. Since abortion is illegal in Bangladesh except to save woman’s live, unsafe and untimely MR remains a major maternal health problem. To better understand this context, in this study we assessed the prevalence and identified factors associated with MR among ever-married women in Bangladesh using data from BDHS 2014. 8084 ever-married women aged 15–49 years were asked whether they have ever used MR service. This question was asked women who had ever heard about MR.

One in every eight ever-married women had MR in their reproductive age in Bangladesh. In contrast to women from Dhaka division, women from Chittagong and Sylhet divisions were less likely to have MR. However, women were more likely to have MR if they were from high SES group, being employed, being parous, and having membership of NGO.

In conclusion, MR is prevalent among Bangladeshi women and independently associated with geographic location, SES, parity, employment and NGO membership status. Health policy should prioritize in reducing spatial and socioeconomic inequalities in relation to MR services by ensuring accessibility and availability of MR services, especially in suburban divisions. Furthermore, abortion should be legalized in Bangladesh that will ultimately reduce the morbidity and mortality associated with unsafe abortion.

## Background

In developing countries, abortion is a significant cause of maternal morbidity and mortality, and often unsafe [1]. As of 2010–2014, an estimated of 55.9 million abortions occur each year globally of which 88% are from developing countries [2] . Between 1990–1994 and 2010–2014, the proportion of abortion has increased from 76 to 88% in developing regions, and 49% of these were unsafe [2, 3]. The risk of unsafe clandestine abortion, related complications and deaths are usually high in the countries where the law prohibits abortion or abortion is only permitted to save the life of a woman [2, 4, 5]. The socio-economic and psychological impact of abortion is also substantial [5].

Bangladesh has a restrictive abortion law, that means abortion is illegal in Bangladesh unless it is necessary for saving life of a woman [6]. However, abortion-related deaths and complications remain a major maternal health problem in Bangladesh though the overall maternal mortality has declined considerably in the last two decades [5]. The incidence of abortion and proportion of abortion-related maternal deaths has increased from 2010 to 2016 in Bangladesh [2, 7–9] . The abortion ratio (the number of abortions per 100 live births) has increased from 18 in 2010 to 35.5 in 2014 while the proportion of abortion-related maternal deaths has increased from 1% in 2010 to 7% in 2016 [7–9]. In 2014, about 1,194,000 induced abortions occurred in Bangladesh, and most of them were not attended by skilled personnel and were conducted in unsafe conditions, which causes severe medical complications including incomplete abortion, hemorrhage, cervical lacerations, sepsis, uterine perforation, bladder injury and shock [10, 11].

Abortion is illegal except to save woman’s live and thus Bangladesh is offering MR services since the independence of the country. The country has included MR in the national family planning programme in 1979 and instructed doctors, paramedics, and family welfare visitors (FWVs) to provide MR services in all government hospitals and in health and family planning complexes as well as government approved private clinics and hospitals. The Government has officially recognized MR as the “procedure of regulating the menstrual cycle when menstruation is absent for a short duration” and this short period has been operationalized “up to 12 weeks after a woman’s last menstrual period if provided by a physician, and up to 10 weeks if provided by a FWV, a type of community-level provider” [12, 13].

The Government of Bangladesh has undertaken significant initiatives to improve the accessibility of MR services. These include training of existing mid-level providers such as nurses and midwives, recruiting more MR service providers, making MR services free of cost, increasing the time frame for allowing MR, developing national MR guidelines on service provision and quality of care, and introducing medicinal MR [11, 14, 15]. However, a recent study based on health facilities and health professionals’ surveys have reported that the rate of MR has declined, and rate of abortion has increased [7, 14]. This facilities and providers-based survey has limitations as the providers often underreport MR procedures that do not fully comply with government regulations [7, 16, 17]. Moreover, it may not estimate the true prevalence because women always do not take MR services from the health facilities, and it could be self-induced or by unskilled providers due to social stigma and denial of MR services by health facilities [16–18]. At the same time, these facilities and providers-based surveys are providing little insight regarding the demand side issues, i.e., what factors are influencing the demand of the MR services among women.

### Objective of the study

Thus, this study aimed to know the proportion of reproductive aged ever-married women who have used MR service and identify the determinants of accessing MR service in Bangladesh using a nationally representative sample from household survey. The findings of this study are important for health policy implications as it can help to design increasing use of MR services, which can reduce maternal morbidity and mortality in Bangladesh.

## Methods

### Study design and sample

The study has extracted data from the latest nationally representative BDHS 2014. The survey followed a two-stage stratified sampling procedure. The survey collected data from 17,863 ever-married women of reproductive age (15 to 49 years) covering rural and urban areas in all administrative regions of the country. Details of sampling are available in the BDHS 2014 final report [19]. However, this study used a sub-sample of 8430 ever-married women aged 15–49 years extracted from survey sample of 17,863 who had ever heard about MR from the original data set of the BDHS 2014. The weighted sample was 8084 ever-married women of reproductive age after adjustments for survey sampling weights and survey design characteristics.

### Data collection

The BDHS survey is conducted by the National Institute for Population Research and Training (NIPORT), Macro International, ICF international, Mitra and Associates, US Agency for International Development (USAID) and Ministry of Health and Family Welfare, Bangladesh. The survey collected information on basic socio-demographic profile and several emerging issues such as marriage, fertility, fertility preference, pregnancy, family planning, maternal health care services utilization, child mortality, knowledge and attitude towards HIV/AIDS and other sexually transmitted infections (STIs) from ever-married women.

### Outcome variable

The BDHS 2014 collected data on the following question: have you ever used MR? This question was asked to those women who have ever heard of MR. The outcome variable was thus defined as ever use of MR services with a binary response (Yes/No).

### Explanatory variables

The wealth index was constructed from data on household durable and non-durable assets (e.g., televisions, bicycles, sources of drinking water, sanitation facilities and construction materials). To create the wealth index, each household asset was assigned a weighted score through principal component analysis and then divided the score distribution into five quintiles equally expressing as wealth quintiles from 1 (lowest) to 5 (highest). Wealth index is used as a background variable to assess demographic and health outcomes of SES. However, the details of the measurement of the wealth index have been provided in the survey report [19]. About 90% of the population belong to the Islam in Bangladesh [19]. Thus, we classified religion as Muslim versus non-Muslim. Women in government or non-government service, day labor, business, domestic service were considered as employed. If a woman was involved in any of the five major NGOs in Bangladesh was considered as NGO member. A woman’s empowerment was defined based on four questions associated with her involvement in the decision-making process about her own and children’s health care seeking behavior, the decision to visit relatives and other family, and decision to buy large household goods, e.g., house or/and land. If a woman has no involvement in decision-making process of these areas was considered that she was not empowered, if she took decision jointly with her husband or other family members was considered moderately empowered, and if she took decision independently was considered that she was highly empowered.

### Statistical analysis

The prevalence estimates and 95% confidence interval (95% CI) for ever used of MR are presented. The estimation with relative standard errors of more than 25% was not considered statistically reliable. Univariate and multivariate mixed-effect logistic regression were used to estimate the odds ratios (OR) and 95% CI. We considered 12 variables as potential explanatory factors for MR. However, age, desire of children, and methods of contraceptive use were excluded from the multivariate model due to multicollinearity with parity. We also excluded religion and level of education from the multivariate model as their *p*-values were > 0.3 in the simple logistic regression analysis. We did not include unwanted pregnancy as a risk factor in our model as the BDHS 2014 asked women whether their last pregnancy was unwanted or not while the question for the outcome of ever use of MR was a lifetime phenomenon. Therefore, division, place of residence, wealth quintile, employment status, parity, NGO membership and women’s empowerment were controlled for the adjusted multivariate mixed-effect logistic model. The multilevel modeling accounts for potential between-clusters variations. All statistical tests were two-sided, and a *p*-value < 0·05 was considered statistically significant. All analyses were performed using statistical software packages Stata (version 15·0; Stata Corp LP, College Station, Texas).

## Results of the study

### Characteristics of the study women

Of 8084 study women, 62.0% lived in rural areas; 20.7% was in the highest wealth quintile, and 89.6% was Muslim (Table 1). The mean (standard error) age at first marriage of the women was 16.2 (0.04) years. About 16.0% of study women had no formal education, and 65.0% were unemployed. Among the study women, 36.2% women reported that they did not use any method of contraception. 91.3% of women had at least one child while 30.6% of women had a desire for more children. Among the participants, 33.2% of women had a membership at any NGOs; and 11.6% women had no decision-making power in the household.

**Table 1:** Weighted prevalence of menstrual regulation by socio-demographics

Characteristics	Frequency (%)	Menstrual Regulation % (95% CI)
Overall	8084	12.3 (11.1–13.4)
Division		
Barisal	619 (7.7)	10.8 (8.8–12.7)
Chittagong	1576 (19.5)	10.2 (8.1–12.3)
Dhaka	2638 (32.6)	15.1 (12.5–17.7)
Khulna	637 (7.9)	10.5 (8.0–13.1)
Rajshahi	1071 (10.2)	10.7 (8.8–12.6)
Rangpur	1241 (15.4)	13.0 (10.3–15.8)
Sylhet	302 (3.7)	7.2 (4.5–9.9)
Place of residence		
Rural	5015 (62.0)	11.5 (9.9–13.1)
Urban	3069 (38.0)	13.5 (12.1–15.0)
Wealth quintile		
Poorest	1571 (19.4)	10.5 (9.1–12.1)
Poorer	1589 (19.7)	10.8 (9.4–12.5)
Middle	1577 (19.5)	11.4 (9.1–13.8)
Richer	1672 (20.7)	13.9 (11.9–15.9)
Richest	1675 (20.7)	14.4 (12.7–16.1)
Religion		
Non-Muslim	839 (10.4)	11.2 (8.5–13.9)
Muslim	7245 (89.6)	12.4 (11.2–13.6)
Age		
< 20	686 (8.5)	3.1 (1.6–4.6)
20–29	3105 (38.4)	9.7 (8.3–11.1)
30+	4293 (53.1)	15.6 (14.0–17.2)
Age at first marriage (Mean ± SE)	16.2 ± 0.04	
Level of education		
No education	1295 (16.0)	12.9 (10.4–15.5)
Primary	2099 (26.0)	12.2 (10.3–14.1)
Secondary	3490 (43.2)	12.3 (10.9–13.7)
Higher	1200 (14.8)	11.5 (9.3–13.7)
Employment status		
No	5254 (65.0)	11.4 (10.1–12.7)
Yes	2826 (35.0)	13.9 (12.2–15.5)
Method of contraceptive use		
Not use	2930 (36.2)	10.2 (8.8–11.6)
Traditional method	737 (9.1)	16.9 (12.3–21.4)
Modern method	4417 (54.6)	12.9 (11.5–14.2)
Desire for more children		
Wants no more	4495 (58.0)	14.4 (13.1–15.7)
Wants more	2370 (30.6)	8.4 (6.6–10.1)
Others	885 (11.4)	14.5 (11.0–18.4)
Parity		
No	704 (8.7)	6.6 (4.4–8.7)
1–2	4700 (58.1)	11.4 (11.0–12.5)
3–4	2252 (27.9)	15.4 (13.5–17.3)
4+	428 (5.3)	14.7 (11.7–18.4)
NGO membership		
No	5397 (66.8)	11.4 (10.3–12.4)
Yes	2687 (33.2)	14.1 (11.9–16.3)
Women’s empowerment		
Not empowered	895 (11.6)	9.8 (8.1–12.0)
Moderately empowered	4467 (58.0)	13.0 (11.3–14.8)
Highly empowered	2337 (30.4)	12.9 (11.3–14.5)

*CI* Confidence Interval, *NGO* Non-Government Organization, *SE* Standard Error

### Prevalence of menstrual regulation

The prevalence of MR is presented in Table 1. The overall prevalence of MR was 12.3% (95% CI: 11.1–13.4%) among (991/8084) ever-married women in Bangladesh. Among the administrative divisions of Bangladesh, the highest and lowest prevalence rates of MR were in Dhaka (15.1%, 95% CI: 12.5–17.7%) and Sylhet (7.2%, 95% CI: 4.5–9.8%), respectively while other divisions except Rangpur had approximately same prevalence rates. MR rate in an urban setting (13.5%, 95% CI: 12.1–15.0%) was higher compared to the rural setting (11.5%, 95% CI: 9.9–13.1%). The prevalence of MR among women with different wealth quintiles revealed that prevalence rates increased with the increase of wealth index among women. The prevalence of MR also increased with increasing age of women, where highest prevalence in older age group (30+ years) was 15.6% (95% CI: 14.0–17.2%) and lowest in younger age group (< 20 years) was 3.1% (95% CI: 1.6–4.6%). The proportion of MR was higher in employed women (13.9%, 95% CI: 12.2–15.5%) compared to unemployed women (11.4%, 95% CI: 10.1–12.7%). Women who used the traditional contraception method had a higher prevalence of having MR (16.9%, 95% CI: 12.3–21.4%) compared with women who did not use any method (10.2%, 95% CI: 8.8–11.6%) or the women who used a modern method (12.9%, 95% CI: 11.5–14.2%). The prevalence of MR among women who had a membership at NGOs and highly empowered in the household was 14.1% (95% CI: 11.9–16.3%) and 12.9% (95% CI: 11.3–14.5%), respectively.

### Factors associated with menstrual regulation

Table 2 presents the unadjusted and adjusted OR with 95% CI obtained from the mixed-effect binary logistic regression model. Compared to Dhaka division, women from Chittagong (AOR 0.74, 95% CI: 0.57–0.96; *p* = 0.026) and Sylhet (AOR 0.53, 95% CI: 0.36–0.77; *p* = 0.001) divisions were less likely to have MR (Table 2). Compared with women in the middle wealth quintile, women in the high (AOR 1.47, 95% CI: 1.18–1.83; *p* = 0.001) wealth quintile as well as in the highest (AOR 1.62, 95% CI: 1.27–2.05; *p* < 0.001) had increased risk of having MR. The employed women were more likely to have MR (AOR 1.35, 95% CI: 1.16–1.56; *p* < 0.001) than unemployed women. Women who had one or two children (AOR 1.73, 95% CI: 1.24–2.40, *p* = 0.001) and ≥ 3 children (AOR 2.56, 95% CI: 1.82–3.58; *p* < 0.001), were more likely to have MR compared with women who had no child. The likelihood of MR was higher for women who had NGO membership (AOR 1.18, 95% CI: 1.02–1.38; *p* = 0.030) than women who had no NGO membership. The risk of having MR was not significantly different between empowered and not empowered women.

**Table 2:** Mixed-effects logistic regression for predictors of menstrual regulation

Characteristics	Menstrual Regulation			
	Unadjusted OR, 95% CI	*p* values	Adjusted OR, 95% CI	*p* values
Division				
Barisal	0.78 (0.59–1.02)	0.074	0.95 (0.72–1.26)	0.731
Chittagong	0.67 (0.51–0.88)	0.003	0.74 (0.57–0.96)	0.026
Dhaka	1.00		1.00	
Khulna	0.77 (0.58–1.02)	0.074	0.86 (0.64–1.15)	0.314
Rajshahi	0.73 (0.56–0.96)	0.002	0.87 (0.66–1.13)	0.301
Rangpur	0.97 (0.76–1.24)	0.789	1.22 (0.95–1.58)	0.123
Sylhet	0.48 (0.33–0.70)	0.000	0.53 (0.36–0.77)	0.001
Place of residence				
Rural	1.00		1.00	
Urban	1.37 (1.18–1.60)	0.000	1.17 (0.98–1.39)	0.084
Wealth quintile				
Lowest	0.87 (0.69–1.08)	0.218	0.82 (0.65–1.04)	0.111
Low	0.87 (0.69–1.09)	0.228	0.88 (0.70–1.11)	0.289
Middle	1.00		1.00	
High	1.34 (1.09–1.66)	0.006	1.47 (1.18–1.83)	0.001
Highest	1.43 (1.16–1.77)	0.001	1.62 (1.27–2.05)	0.000
Religion				
Non-Muslim	1.00			
Muslim	1.09 (0.86–1.39)	0.472		
Age				
< 20	1.00			
20–29	3.03 (1.98–4.65)	0.000		
≥ 30	5.32 (3.50–8.08)	0.000		
Level of education				
No education	1.00			
Primary	0.98 (0.79–1.21)	0.831		
Secondary	0.96 (0.79–1.18)	0.704		
Higher	0.93 (0.73–1.19)	0.560		
Employment status				
No	1.00		1.00	
Yes	1.32 (1.15–1.52)	0.000	1.35 (1.16–1.56)	0.000
Method of contraceptive use				
Not use	1.00			
Traditional method	1.49 (1.17–1.90)	0.001		
Modern method	1.28 (1.10–1.48)	0.001		
Desire for more children				
Wants no more	1.00			
Wants more	0.52 (0.43–0.61)	0.000		
Others	1.04 (0.73–1.11)	0.324		
Parity				
No	1.00		1.00	
1–2	2.03 (1.48–2.78)	0.000	1.73 (1.24–2.40)	0.001
3+	2.76 (2.00–3.80)	0.000	2.56 (1.82–3.58)	0.000
NGO membership				
No	1.00		1.00	
Yes	1.24 (1.07–1.42)	0.003	1.18 (1.02–1.38)	0.030
Women’s empowerment				
Not empowered	1.00		1.0	
Moderately empowered	1.43 (1.13–1.81)	0.003	1.14 (0.90–1.45)	0.282
Highly empowered	1.39 (1.09–1.79)	0.008	1.19 (0.93–1.53)	0.167
Variance component	–		0.14 (0.06–0.29)	0.001

*p < 0.05, **p < 0.01, ***p < 0.001

*CI* Confidence Interval, *NGO* Non-Government Organization, *OR* Odds Ratio

## Discussion

This nationally representative study demonstrates that one in every eight women had MR in their reproductive age in Bangladesh. The MR was highly prevalent amongst women aged more than 20 years old. The significant determinants of MR were geographic region, SES, employment, parity and NGO membership status.

The proportion of MR was disproportionate with the regional concentration, which showed that women from Sylhet and Chittagong division were less likely to have MR compared with women from Dhaka division. In contrast to women from Dhaka division, women in Sylhet and Chittagong may have less knowledge and access to MR services, and they face higher restriction and social stigma, which are consistent with previous studies [4, 20, 21]. In addition, MR services are available in the capital city of Bangladesh due to the highest concentration of most of the public and private clinics, hospitals, and NGOs than other divisions. However, the Union Health and Family Welfare Centres (UH & FWCs) and NGOs provide MR services particularly in the sub-urban and rural areas, and only about half of the all UH and FWCs are capable of providing MR services in Bangladesh, which has precipitous decline accounted to three-quarters of the national decline between 2010 and 2014 [7, 11, 22]. Evidence also suggests that social or religious factors, conservative health beliefs, reluctance to perform MR, lack of proper training, sufficient stuff or equipment and space could explain the sharp decline of MR services, especially in the UH and FWCs. Furthermore, religious or social reasons (43%), health beliefs (32%) and reluctance to perform MR (24%) were mentioned by about half of the UH and FWCs officials as the reasons of this deterioration [11, 23]. Hence, clandestine abortions may become choice for the women, which are mostly unsafe and increase abortion complications as well as burden related to abortions [3].

Consistent with studies conducted in developing countries, our study revealed that women with higher SES were more likely to have MR than their counterparts [4, 21, 24–29]. Women from higher SES have more control over their reproductive behavior, access to better living standard and health care particularly both public and private health care services [27]. In contrast to our results, some studies showed a reverse association between higher SES of women and lower risk of MR [30, 31]. However, most of these studies were conducted in developed countries in which structural mechanisms are entirely different from developing countries [30, 32].

Employed women had higher odds of MR than the unemployed women, which is consistent with previous literature [24, 26, 29, 33]. The possible explanation could be the overall empowerment of women, which enable them to act on fertility choice, traditional role of mothers, reduce the family size, avoidance of unintended pregnancy and access to MR services [4, 22–24, 26, 28, 29, 33].

Inconsistent with other studies [24, 30] education was not associated with MR in our unadjusted model, which has been omitted from the final model. In Bangladesh, education is merely associated with income generating activities or healthcare decision-making process. For instance, a large number of women with no education working in garments industries while many highly educated women are not in formal employment and involve only in household duties [34]. Results indicate that parity or number of the children was positively associated with the MR. This evidence is consistent with the previous study, which also argued that the number of living children or parity are associated with higher likelihood seeking MR services in Bangladesh [30]. The plausible mechanism could be the use of MR services to limit or delay births for reducing their family size, which is highly prevalent in the South Asian countries [35]. The NGO membership of women had a positive effect on the likelihood of having MR compared with the non-membership status of the women. The reason could be their empowerment, participation in income generating activities, social network and access to MR services. For instance, Bangladesh Rural Advancement Committee, the largest NGO in the world, works for improving reproductive health in Bangladesh.

In line with a previous study [32], moderate and higher level of women empowerment were positively associated with the likelihood of MR in the initial analysis, but significance was disappeared after adjusting other determinants. The plausible reason could be the prevailing patriarchal social structure and husband’s control over women’s healthcare decision as they are the key decision maker in the South Asian context, particularly in Bangladesh [32, 36]. Consequently, even empowered women decide about their own healthcare jointly with husband or by someone else. For example, family member of husband such as in-laws may have an influence on the decision making process of women’s use of MR due to the existing sociocultural settings [32, 36, 37].

### Strength and weakness

The strengths of this study were using the recent and large nationally representative database, with a high response rate (98%). Secondly, the present study used multilevel modelling for adjusting cluster variations. The study acknowledges some potential limitations as well. For example, we cannot infer a causal relationship between determinants and MR due to the cross-sectional nature of the data. Moreover, the inclusion of all potential explanatory variables might introduce table two fallacy. Although unintended pregnancy is the root cause of MR, we were unable to use this variable in our model due to the lack of precise data. Since the BDHS collected relevant data from ever-married women only, this is a limitation for not capturing information of MR that might happen to any unmarried women or pregnancy outside of marriage that might underestimate the true prevalence of MR.

## Conclusions

MR is prevalent in Bangladeshi women and independently associated with geographic location, SES, employment, parity and NGO membership status. Health policy should prioritize in reducing spatial and socioeconomic inequalities in relation to MR services by ensuring accessibility and availability of MR services, especially in suburban divisions. Furthermore, abortion should be legalized in Bangladesh that will ultimately reduce morbidity and mortality associated with unsafe abortion.

## Data Availability

The BDHS data were obtained from the MEASURES DHS. The data is available on the website of the global DHS program, which could be accessed after getting registered with the MEASURES DHS. Details: https://dhsprogram.com/data/Using-DataSets-for-Analysis.cfm

## Abbreviations

AOR: Adjusted Odds Ratios
BDHS: Bangladesh Demographic and Health Survey
CI: Confidence Interval
DHS: Demographic and Health Survey
FWVs: Family Welfare Visitors
IRB: Institutional Review Board
MR: Menstrual Regulation
NGO: Non-government Organization
NIPORT: National Institute for Population Research and Training
OR: Odds Ratios
SES: Socioeconomic Status
STIs: Sexually Transmitted Infections
UH & FWCs: Union Health and Family Welfare Centres
USAID: US Agency for International Development
